# PEI-Fe_3_O_4_/PTA-AuNPs Hybrid System for Rapid DNA Extraction and Colorimetric LAMP Detection of *E. faecium*

**DOI:** 10.3390/bios15090601

**Published:** 2025-09-12

**Authors:** Muniyandi Maruthupandi, Haang Seok Choi, Nae Yoon Lee

**Affiliations:** 1 Department of BioNano Convergence, Gachon University, 1342 Seongnam-daero, Sujeong-gu, Seongnam-si 13120, Gyeonggi-do, Republic of Korea; maruthu1328@gachon.ac.kr; 2 Department of BioNano Technology, Gachon University, 1342 Seongnam-daero, Sujeong-gu, Seongnam-si 13120, Gyeonggi-do, Republic of Korea; choihas@gachon.ac.kr

**Keywords:** polyethylenimine, Fe_3_O_4_, LAMP, tannic acid, AuNPs, colorimetry, *E. faecium*

## Abstract

This study introduces a novel nucleic acid testing (NAT) protocol that integrates rapid deoxyribonucleic acid (DNA) extraction, isothermal amplification, and visual detection to enable efficient analysis of opportunistic pathogens. Polyethylenimine-functionalized iron oxide (PEI-Fe_3_O_4_) nanoparticles were prepared by combining PEI, acting as a stabilizing agent, with iron salt, which was utilized as the metal ion precursor by the ultrasonication-assisted co-precipitation method, and characterized for structural, optical, and magnetic properties. PEI-Fe_3_O_4_ exhibited cationic and anionic behavior in response to pH variations, enhancing adaptability for DNA binding and release. PEI-Fe_3_O_4_ enabled efficient extraction of *E. faecium* DNA within 10 min at 40 °C, yielding 17.4 ng/µL and achieving an extraction efficiency of ~59% compared to a commercial kit (29.5 ng/µL). The extracted DNA was efficiently amplified by loop-mediated isothermal amplification (LAMP) at 65 °C for 45 min. Pyrogallol-rich poly(tannic acid)-stabilized gold nanoparticles (PTA-AuNPs) served as colorimetric probes for direct visual detection of the DNA amplified using LAMP. The magnetic-nanogold (PEI-Fe_3_O_4_/PTA-AuNPs) hybrid system achieved a limit of quantification of 1 fg/µL. To facilitate field deployment, smartphone-based RGB analysis enabled quantitative and equipment-free readouts. Overall, the PEI-Fe_3_O_4_/PTA-AuNPs hybrid system used in NAT offers a rapid, cost-effective, and portable solution for DNA detection, making the system suitable for microbial monitoring.

## 1. Introduction

Rapid and reliable detection of bacteria is essential in diagnostics, food safety, and environmental monitoring, since bacterial infections pose a major concern to public health worldwide. Conventional nucleic acid testing (NAT) methods, including loop-mediated isothermal amplification (LAMP), polymerase chain reaction (PCR), and commercial extraction kits, offer high sensitivity and accuracy, but are limited by high cost, long process time, and high-cost equipment, restricting their suitability for point-of-care testing (POCT). To overcome these limitations, nanomaterial-assisted strategies have gained increasing attention in the development of biosensors. In particular, magnetic nanoparticles (NPs) such as magnetite iron oxide (Fe_3_O_4_) enable rapid deoxyribonucleic acid (DNA) extraction and separation, minimize sample interference, and enhance downstream assay performance [1]. Antibody-free approaches, such as the use of polymer-functionalized NPs (e.g., polyethylenimine (PEI), chitosan, and polyamines), provide simple and cost-effective means for binding DNA through electrostatic interaction, thus reducing assay complexity [2,3,4,5,6,7,8].

Commercial DNA extraction methods, such as silica spin columns, phenol–chloroform, cetyltrimethylammonium bromide, and Chelex resin, have limitations following low DNA yield, residual impurities, lengthy protocols, high-temperature requirements, and toxic reagents [9]. NPs are ultra-small materials characterized by quantum confinement effects and a high surface-to-volume ratio, which exhibit novel properties, such as surface plasmon resonance and magnetic responsiveness. These remarkable features enable a wide range of applications in medicine, targeted drug delivery, biosensors, DNA extraction, and environmental monitoring, offer enhanced selectivity and efficiency [10]. Magnetic solid-phase extraction presents significant advantages over alternative strategies, such as rapidity, simplicity, low-temperature requirements, low cost, and enhanced purity. Magnetic NPs (MNPs) are easily dispersed in aqueous or buffer solutions and are efficiently separated by applying an external magnetic field. This facilitates the efficient adsorption and desorption of DNA, primarily driven by electrostatic interactions between the negatively charged DNA and the positively functionalized surface of the MNPs [11,12].

Polymers are widely used to enhance the performance of MNPs in DNA extraction and cell lysis. The abundance of primary (–NH_2_), secondary (–NH–), and tertiary (–N–) amine groups in PEI results in a very effective cationic polymer. Because amine groups enable strong electrostatic interactions with the negatively charged DNA, PEI is well-suited for enhancing cell lysis and facilitating DNA extraction from complex bacterial samples. PEI can change between cationic and anionic forms in response to pH variation, which enhances its adaptability to different extraction scenarios. PEI also acts as a stabilizer and reducing agent for the preparation of NPs [13,14]. Previous studies have explored charge-switching DNA extraction methods driven by pH-sensitive electrostatic interactions. For example, Nakagawa et al. fabricated a microchip modified with aminosilane. On the channel surface, positively charged amine groups extracted DNA under acidic and alkaline pH variations. This method allowed for DNA purification from whole blood while being compatible with PCR, even though harsh alkaline elution conditions limited its PCR readiness and reduced DNA recovery efficiency (27–40%) [15]. Similarly, Cao et al. reported that chitosan-coated silica beads and microchips could capture and release DNA in a pH-dependent manner. At acidic pH 5, DNA strongly bound to the protonated chitosan surface, whereas at mildly alkaline pH 9, it was quickly released. This method showed high extraction efficiency (up to 92%) and did not use chaotropic salts for lysis [16].

Moreover, the naked eye for NAT focuses on developing sensing methods that are rapid and accurate. The LAMP method for amplifying nucleic acids is a promising new tool for quick medical tests, because it is specific, quick, and can function well at low temperatures, at approximately 65 °C. Recent studies have explored the use of noble metal NPs, such as Pt, Au, Ag, and Pd, in colorimetric assays, due to their unique optical and catalytic properties, which enable the visual detection of LAMP-amplified DNA through observable color changes. Tannic acid (TA) acts as a self-reducing agent and stabilizer due to its high polyphenol content and polymeric nature. DNA serves as a counter-ion, binding metal ions through noncovalent interactions to neutralize the phosphate backbone. Consequently, nitrogenous bases in the DNA reduce gold salt to gold NPs (AuNPs). The AuNPs can be easily visualized owing to their color change, providing a simple and effective method for detecting DNA amplification [17,18,19].

Building on these advances, this study introduces PEI-functionalized Fe_3_O_4_ (PEI-Fe_3_O_4_) NPs via a simple co-precipitation method. The structural and optical properties of PEI-Fe_3_O_4_ were confirmed using various characterization techniques. PEI-Fe_3_O_4_ was used for rapid and efficient DNA extraction from one of the major opportunistic pathogens, *Enterococcus faecium* (*E. faecium*). Subsequently, the extracted DNA was used for a LAMP reaction. The resulting LAMP-amplified DNA was visually detected using poly(tannic acid)-stabilized AuNPs (PTA-AuNPs) as a colorimetric probe. Our magnetic-nanogold (PEI-Fe_3_O_4_/PTA-AuNPs) hybrid system achieves DNA extraction and detection within 70 min with a cost of only $1.5/test. The developed PEI-Fe_3_O_4_/PTA-AuNPs system offers a rapid, low-cost, and equipment-free approach for bacterial cell lysis, DNA extraction, and visual detection of DNA, thereby enabling precise pathogen identification and extending applicability to fields such as genetic disorders, oncology, and virology.

## 2. Materials and Methods

### 2.1. Reagents and Chemicals

Sodium chloride (NaCl, ≥99%), iron(III) chloride (FeCl_3_, ≥98%), iron(II) chloride (FeCl_2_, ≥98%), gold(III) chloride trihydrate (HAuCl_4_·3H_2_O, ≥99.99%), and tannic acid (TA) (≥98%) were bought from Sigma-Aldrich (St. Louis, MO, USA). Ethanol (EtOH), sodium hydroxide (NaOH), and polyethylene glycol (PEG, Mw ~1000 g/mol) were bought from Daejung Chemicals & Metals (Siheung-si, Gyeonggi-do, Republic of Korea). An ammonia solution (NH_4_OH, 25%) was bought from Merck (Rahway, NJ, USA). PEI (≥99%, Mw ~600 g/mol) was bought from Thermo Fisher Scientific (Waltham, MA, USA). A 1× phosphate-buffered saline (PBS), a 10× tris-acetate-ethylenediaminetetraacetic acid (TAE) buffer, and a 1× TAE buffer were acquired from Biosesang Co., Ltd. (Yongin-si, Gyeonggi-do, Republic of Korea).

The LAMP kit contains 100 mM of magnesium sulfate (MgSO_4_), a 10× isothermal amplification buffer (ITB), and Bst 2.0 WarmStart DNA polymerase, which was bought from New England BioLabs (Ipswich, MA, USA). Deoxyribonucleoside triphosphate (dNTPs) and agarose powder were acquired from BioFACT (Daejeon, Republic of Korea). A 100 bp DNA ladder was bought from Takara (Shiga, Japan). Ethidium-bromide-based DNA intercalation dye (Loading STAR) was bought from DyneBio (Seongnam-si, Gyeonggi-do, Republic of Korea). The plasmid DNA preparation kit (Hi Gene) was bought from BioFACT (Daejeon, Republic of Korea). Primer DNA *E. faecium* and *pneumolysin* (*ply*) gene of *Streptococcus pneumoniae* (*S. pneumoniae*) was acquired from Cosmo Genetech Co., Ltd. (Seoul, Republic of Korea). A neodymium (NdFeB) super-strong magnet bar was bought from JS Magnet (Incheon, Republic of Korea). The real sample analysis was performed using river water (RW) samples collected from the Jungnangcheon Stream (Yangju, Gyeonggi-do, Republic of Korea).

### 2.2. Synthesis of Fe_3_O_4_ and PEI-Fe_3_O_4_

Figure S1 presents a simplified synthesis procedure for Fe_3_O_4_ and PEI-Fe_3_O_4_ using an ultrasonication-assisted co-precipitation method. Initially, 0.5 g of FeCl_2_ and 1.2 g of FeCl_3_ were dissolved in 35 mL of deionized (DI) water in a 250 mL flask. The mixture was sonicated for 15 min in an ultrasonic bath. Subsequently, 2 mL of 30 mM NH_4_OH was added to the beaker, followed by 15 min of sonication. A color change from yellowish-brown to black signified the formation of Fe_3_O_4_. The Fe_3_O_4_ precipitate was purified by centrifugation, washed using DI water, and dried at 65 °C for 24 h. The functionalization of Fe_3_O_4_ with PEI was accomplished using a similar sonication-assisted technique. Briefly, 0.6 g of Fe_3_O_4_ was dispersed in 20 mL of 70% EtOH in a 250 mL beaker. The mixture was sonicated for 5 min in an ultrasonic bath. Subsequently, 20 mL of 5 mM PEI solution was added to the same beaker containing the Fe_3_O_4_ dispersion, followed by sonication for an additional 15 min. A color change from black to dark black shows the formation of PEI-Fe_3_O_4_. The precipitate PEI-Fe_3_O_4_ was collected and purified by centrifugation, washed with DI water, and dried at 65 °C for 24 h. The resulting Fe_3_O_4_ and PEI-Fe_3_O_4_ samples were used for further characterization and application studies.

**Scheme 1:**
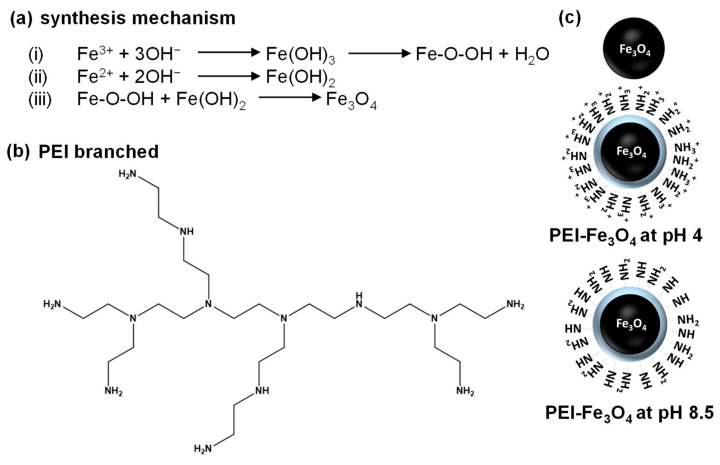
(**a**) Synthesis reaction mechanism of magnetite iron oxide (Fe_3_O_4_); (**b**) molecular structure of polyethylenimine (PEI); (**c**) schematic illustration of the surface state of Fe_3_O_4_ and PEI-functionalized Fe_3_O_4_ (PEI-Fe_3_O_4_) at pH 4 and 8.5.

Scheme 1 illustrates the synthesis and functionalization of Fe_3_O_4_ and PEI-Fe_3_O_4_. Scheme 1a illustrates the reaction mechanism of Fe_3_O_4_, which occurs via the reaction of Fe^3^^+^ and Fe^2^^+^ ions with hydroxide ions (OH^–^) to form iron hydroxides such as Fe(OH)_3_ and Fe(OH)_2_. Subsequently, intermediates combine to form Fe_3_O_4_ by interacting Fe-O-OH with Fe(OH)_2_. Scheme 1b illustrates the chemical structure of branched PEI, a polymer characterized by numerous amine (–NH_2_, –NH–, –N–) groups that facilitate strong interaction with NPs surfaces through electrostatic and polymeric interactions. Scheme 1c illustrates unmodified Fe_3_O_4_ and PEI-Fe_3_O_4_ at different pH values. At pH 4, the amine groups of PEI become protonated (–NH_3_^+^), leading to increased positive surface charge. In contrast, at pH 8.5, the amine groups remain largely deprotonated (–NH_2_), resulting in a negative surface charge. The pH-responsive behavior of PEI enhances the stability of Fe_3_O_4_ NPs, modulates surface charge, and improves the ability to effectively interact with DNA [20,21,22].

### 2.3. Instruments and Characterizations

The optical properties of PEI, Fe_3_O_4_, and PEI-Fe_3_O_4_ were studied using a UV–Visible/NIR spectrophotometer (V-770, JASCO, Hachioji, Tokyo, Japan) over a range of wavelengths from 200 to 800 nm. Fourier-transform infrared (FT-IR) spectroscopy was performed using an FT/IR-4700 spectrometer (JASCO, Hachioji, Tokyo, Japan) to analyze the functional groups of PEI, Fe_3_O_4_, and PEI-Fe_3_O_4_. High-resolution X-ray diffraction (HR-XRD) patterns were obtained using a SmartLab diffractometer (Rigaku Corporation, Neu-Isenburg, Germany), operating at 40 kV with Cu Kβ radiation (λ = 1.5406 Å), over a 2θ range of 10° to 80° at a scanning speed of 2°/min. The shape and morphology of PEI-Fe_3_O_4_ were studied using high-resolution transmission electron microscopy (HR-TEM), (JEM-F200, JEOL, Akishima, Tokyo, Japan) at a voltage of 300 kV, serviced by the Center for Bionano Materials Research at Gachon University (Seongnam, Republic of Korea). Magnetic properties were analyzed using a vibrating sample magnetometer (VSM), Lake Shore 7407-S Series (Lake Shore Cryotronics, Inc., Westerville, OH, USA), in a magnetic field of up to ±30 kOe at 300 K. DNA amplification was performed using a thermal cycler (Bioer, Hangzhou, Zhejiang, China). The agarose gel electrophoresis images were captured using a UV transilluminator (Korea Labtech, Namyangju-si, Gyeonggi-do, Republic of Korea). DNA was quantified with a spectrophotometer (NanoDrop 2000; Thermo Scientific, Waltham, MA, USA) based on the absorbance at 260 nm.

### 2.4. Preparation of Bacterial Samples

*E. faecium* (ATCC BAA-2127) was cultivated in 5 mL of brain heart infusion (BHI) broth and incubated at 37 °C for 24 h with continuous agitation at 300 rpm in a shaking incubator (Biofree Co., Ltd., Seoul, Republic of Korea). After incubation, the bacterial concentration was determined by counting colony-forming units (6 × 10^6^ CFU/mL) on BHI agar plates.

### 2.5. DNA Extraction Using Commercial Kit, Fe_3_O_4_, and PEI-Fe_3_O_4_

The cultivated *E. faecium* samples were subjected to DNA extraction using a plasmid DNA extraction kit. The extraction was carried out following the protocol using lysis enzymes and proteinase K, with centrifugation steps and incubation at 70 °C for 90 min. Figure S2 illustrates the rapid DNA extraction protocol using PEI-Fe_3_O_4_ via a magnetic separation method, completed within 10 min at an incubation temperature of 40 °C. Briefly, 250 µL (6 × 10^6^ CFU/mL) of *E. faecium* culture was transferred to a 2 mL centrifuge tube, first mixed with 250 µL of PEI-Fe_3_O_4_ suspension (10 mg/mL in PBS, pH 4). The mixture was incubated for 5 min at 40 °C to induce cell lysis. Subsequently, a binding buffer with 1.25 M NaCl and 10% PEG mixture was transferred and maintained at 28 °C for 2 min to facilitate DNA binding. Then, a magnetic bar was used to attract the PEI-Fe_3_O_4_-DNA complex, and any impurities were removed for 2 min. For elution, 100 µL of 10× TAE buffer (pH 8.5) was added to release the bound DNA, followed by magnetic separation to isolate the PEI-Fe_3_O_4_. The DNA-containing supernatant was transferred to a separate 2 mL centrifuge tube for further analysis. A similar procedure was utilized for DNA extraction using unmodified Fe_3_O_4_. The extracted DNA was used for DNA amplification and colorimetric sensing. The DNA concentration was calculated using the absorbance spectrum at 260 nm and confirmed by agarose gel electrophoresis.

**Scheme 2:**
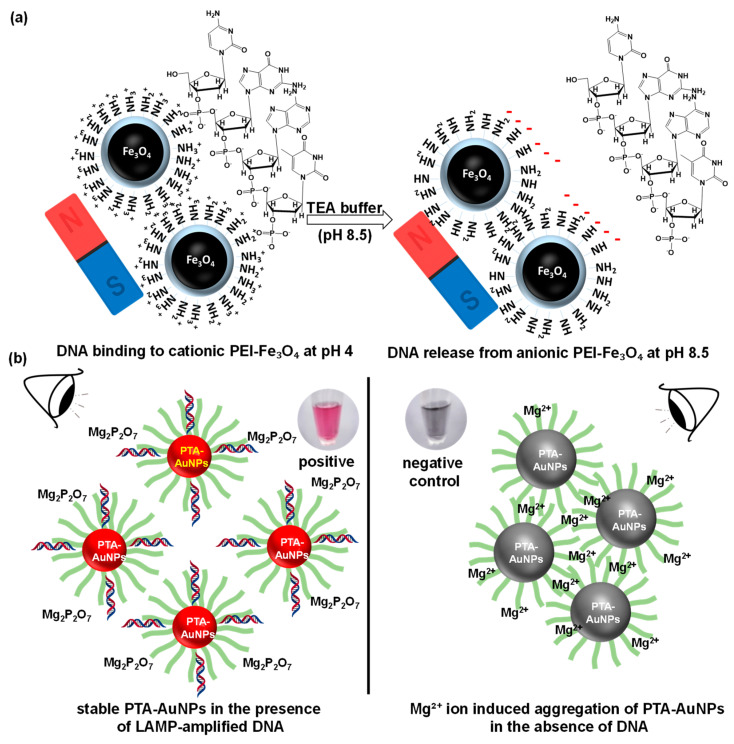
(**a**) Mechanism of DNA binding and release mediated by PEI-Fe_3_O_4_; (**b**) mechanism of colorimetric detection of LAMP-amplified DNA.

Scheme 2a illustrates a mechanism of DNA binding and release with PEI-Fe_3_O_4_. PEI destroys bacteria by breaking down cell walls and acting as a surfactant at 40 °C. Park et al. found that PEI-treated silver NPs (AgNPs) damage bacterial membranes and increase cell breakdown at mild temperatures. The pH-responsive surface properties of PEI-Fe_3_O_4_ play a crucial role in mediating the binding and releasing of DNA after cell lysis. Under acidic conditions (pH 4), the addition of a binding buffer caused the amine groups in PEI to become protonated, resulting in a positively charged NPs surface. DNA is efficiently captured through electrostatic attraction between the positively charged NPs and negatively charged DNA bases (adenine, thymine, guanine, and cytosine). The amine groups are deprotonated by the addition of TAE buffer (pH 8.5), which changes the PEI-Fe_3_O_4_ surface to a negatively charged surface state. This charge reversal induces electrostatic repulsion, thereby releasing bound DNA from the NPs. Thus, a fast and effective method for lysing bacterial cells and extracting DNA is possible with the use of PEI-Fe_3_O_4_ and an external magnet. The reason for choosing Fe_3_O_4_ NPs is that they are highly effective for DNA extraction due to their superparamagnetic behavior, strong DNA-binding efficiency, enhanced lysis, excellent biocompatibility, easy recyclability, and highly modifiable surface chemistry. These properties allow for rapid magnetic separation, high DNA yield, and compatibility with further applications. In contrast, other nanomaterials such as SiO_2_, TiO_2_, AuNPs, and AgNPs lack fundamental magnetic properties and often exhibit cytotoxic effects, which limit their overall efficiency and biocompatibility in DNA extraction processes [21,23,24,25,26].

### 2.6. Primer Design

The plasmid *esp gene* was chosen as a specific marker for identifying *E. faecium*. A set of five primers was designed using the Primer Explorer V5 software (Eiken Co., Ltd., Japan, https://primerexplorer.eiken.co.jp/e/, accessed on 21 July 2025), comprising one loop primer (LB), two outer primers (B3 and F3), and two inner primers (BIP and FIP). These primer sets demonstrated excellent precision and responsiveness for detecting *E. faecium*. These primers were used in LAMP assays, making reliable detection of *E. faecium* in a range of real samples possible. Primer sequences and their specific target regions are listed in Table S1.

### 2.7. Loop-Mediated Isothermal Amplification Assay

The primer mixture was prepared by combining 56 μL of DNA-free water, 16 μL each of inner primers (BIP and FIP), 2 μL each of outer primers (B3 and F3), and 8 μL of loop primer (LB). The LAMP reaction was executed in 25 μL of LAMP mixture at 65 °C for 45 min. To prepare the LAMP reaction mixture, the following reagents were used: 13.5 μL of DNA-free water, 3 μL of isothermal amplification buffer (ITB), 2.5 μL of the prepared primer mix, 3 μL of dNTPs, 1.5 μL of 6 mM MgSO_4_, 1 μL of the target DNA, and 0.5 μL of *Bst* 2.0 WarmStart DNA polymerase. An amount of 1 μL of DNA-free water was added to the negative LAMP control, which did not contain any DNA. After the LAMP reaction, the results were analyzed using gel electrophoresis to confirm the amplification of the target DNA. This study used PTA-AuNPs for colorimetric assessment to visually detect DNA amplification, with signal intensity quantified via smartphone-based color measurement (Figure S2).

### 2.8. Agarose Gel Electrophoresis

Gel electrophoresis was used to confirm the presence of LAMP-amplified DNA. Gels were prepared with a 1.5% agarose solution, consisting of 300 mg of agarose dissolved in 20 mL of 1× TAE buffer for small gel plates and 600 mg in 40 mL of 1× TAE buffer for larger plates. A DNA ladder, negative control (NC), and positive (P) LAMP products were loaded into the wells, mixed with ethidium bromide. Electrophoresis was performed at the same voltage for 20 min. Finally, gel visualization was performed using a UV transilluminator, and the results were stored through image capture.

### 2.9. Colorimetric Detection of LAMP Using PTA-AuNPs

PTA-AuNPs were employed for the visual colorimetric detection of LAMP-amplified DNA. The colorimetry was performed in a 0.5 mL PCR tube, where 5 µL of 5 mM HAuCl_4_ was mixed with 5 µL of 1 mM TA, maintaining the mixture pH at 8.5 with the help of NaOH. Afterwards, 5 µL of the LAMP reaction mixture was injected into the PCR tube. The resulting solution was incubated at 40 °C for 5 min. A distinct color change between the LAMP-NC and P samples was observed, attributed to the stability differences in the PTA-AuNPs. Colorimetric detection based on PTA-AuNPs is a valuable tool for naked-eye diagnostics.

Scheme 2b and Figure S2 present the mechanism of the colorimetric detection of LAMP-amplified DNA, which relies on the formation and stability of PTA-AuNPs at pH 8.5. TA undergoes self-polymerization to form PTA, which acts as an effective stabilizing and reducing agent, because of its deprotonated phenolic groups. TA can produce PTA-AuNPs at a mildly basic pH 8.5 [10]. A large amount of DNA is produced in LAMP-amplified DNA, which interacts with PTA-AuNPs through hydrogen bonding and π–π stacking between the DNA bases and the aromatic groups of PTA. These interactions enhance the colloidal stability of the NPs and prevent their aggregation. Additionally, a portion of the Mg^2^^+^ ions is consumed during the amplification process, forming Mg-pyrophosphate (Mg_2_P_2_O_7_) as a by-product. A low concentration of free Mg^2^^+^ minimizes the potential for the disruption of the stabilizing PTA layer on the AuNPs surface. Consequently, the PTA-AuNPs remain well dispersed, maintaining a red color. In contrast, LAMP-NC samples lack DNA, leaving Mg^2^^+^ ions abundant in the reaction mixture. Excess Mg^2^^+^ can bind with the deprotonated phenolate groups of PTA, creating PTA-Mg^2^^+^ complexes. This lower DNA concentration and complex formation reduce the electrostatic and steric stabilization provided by the PTA layer, promoting AuNPs aggregation. Consequently, the solution exhibits a visible gray color. The colorimetric detection of LAMP-amplified DNA relies on the DNA-mediated stabilization of PTA-AuNPs in P samples. In contrast, the absence of DNA in NC samples leads to magnesium ion (Mg^2^^+^)-induced destabilization, resulting in subsequent NPs aggregation [27,28,29].

### 2.10. Sensitivity and Selectivity Test

The extracted plasmid DNA was used to evaluate the sensitivity of the LAMP assay for colorimetric sensing. From an initial concentration of 10 ng/μL, the plasmid DNA undergoes serial dilutions in DNA-free water to a final concentration of 0.1 fg/μL. The limit of quantification (LOQ) was determined by agarose gel electrophoresis and colorimetric detection. Additionally, 10 ng/μL of *Streptococcus pneumoniae* DNA was used as a cross-interference control for the selectivity study.

### 2.11. Smartphone-Based Detection of LAMP Using PTA-AuNPs

Colorimetric detection of P and NC samples was performed using PTA-AuNPs to assess the sensitivity across varying DNA concentrations. Observable color changes in the PTA-AuNPs solution acted as a visual indicator, enabling distinction between the NC and P samples based on the extent of LAMP. The resulting color changes were captured using a 200-megapixel mobile phone camera. The captured images were analyzed using the Color Picker application (version 7.9.0, Mikhail Gribanov, Ukraine) to obtain the RGB values. Variations in the DNA concentration were estimated by the changes in RGB/R. The limit of detection (LOD) for DNA was estimated using LOD = 3 S/m, where S denotes the standard deviation of the NC (RGB/R), and m represents the slope of the calibration.

## 3. Results and Discussion

### 3.1. Absorption and Vibrational Spectra Analysis of PEI, Fe_3_O_4_, and PEI-Fe_3_O_4_

**Figure 1:**
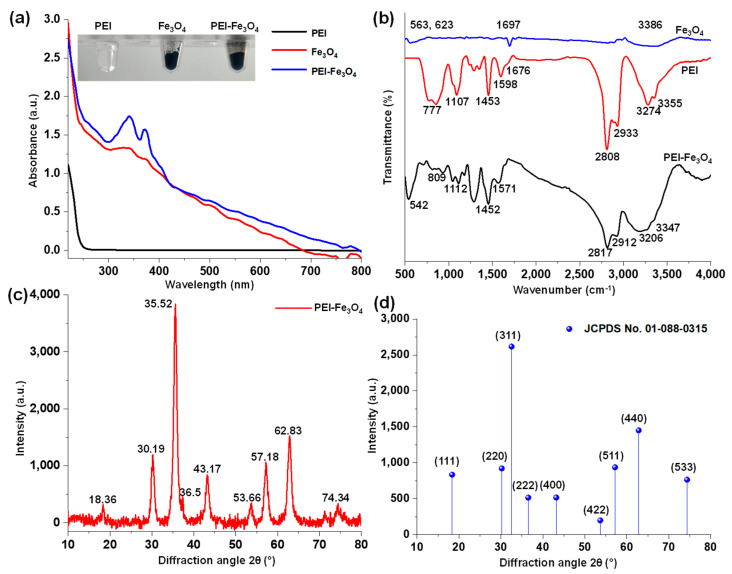
(**a**) UV-Vis spectra of PEI, Fe_3_O_4_, and PEI-Fe_3_O_4_ (inset: corresponding photographs); (**b**) FT-IR spectra of PEI, Fe_3_O_4_, and PEI-Fe_3_O_4_; (**c**) HR-XRD pattern of PEI-Fe_3_O_4_; (**d**) JCPDS pattern of PEI-Fe_3_O_4_.

Figure 1a demonstrates the absorption spectra of PEI, Fe_3_O_4_, and PEI-Fe_3_O_4_. The PEI spectrum shows no observable absorption band, whereas Fe_3_O_4_ displays broad absorption peaks with a maximum around 340, 374, and 408 nm. After the PEI coating, this peak shifts to 339, 370, and 404 nm. Both Fe_3_O_4_ and PEI-Fe_3_O_4_ exhibited characteristic absorption bands associated with surface electronic transitions of Fe_3_O_4_ [30,31]. The inset of Figure 1a presents photographs of the corresponding samples, showing that PEI is colorless in solution, whereas Fe_3_O_4_ and PEI-Fe_3_O_4_ appear as black solids. The formation of Fe_3_O_4_ and PEI-Fe_3_O_4_ was preliminarily confirmed through the presence of the absorption bands and their color.

The FT-IR spectra of PEI, Fe_3_O_4_, and PEI-Fe_3_O_4_ are presented in Figure 1b. The FT-IR spectrum of Fe_3_O_4_ exhibits stretching vibrations of the Fe-O bond observed at 563 and 623 cm^−1^. Moreover, the two peaks at 1697 and 3386 cm^−1^ are attributed to the bending and stretching vibrations of OH groups from adsorbed water [31,32,33]. The FT-IR spectrum of PEI exhibits characteristic peaks corresponding to functional groups of N-H stretching at 777 cm^−1^, C-N bending (CH_2_-NH) at 1107 cm^−1^, CH_2_ scissoring vibrations at 1453 cm^−1^, aromatic N-H bending at 1598 and 1676 cm^−1^, CH stretching at 2808 cm^−1^, CH_2_ bending at 2933 cm^−1^, and NH_2_/NH stretching at 3274 and 3355 cm^−1^. These results confirm the branched structure of PEI, which is consistent with its previously reported FT-IR spectrum [34].

The FT-IR spectrum of PEI-Fe_3_O_4_ showed the following peaks at 3206 and 3347 cm^−1^ (NH_2_/NH stretching), 2912 cm^−1^ (CH_2_ bending), 2817 cm^−1^ (CH stretching), 1517 cm^−1^ (N-H bending), 1452 cm^−1^ (CH_2_ scissoring), 1112 cm^−1^ (C-N bending, CH_2_-NH), 809 cm^−1^ (N-H stretching), and 542 cm^−1^ (Fe-O stretching) [31,32,33,34]. The peak at 542 cm^−1^ confirms the presence of Fe_3_O_4_, whereas the shifts in other functional group frequencies suggest interactions between PEI and the Fe_3_O_4_ surface. The NH groups contribute to the electrostatic and steric stabilization of PEI-Fe_3_O_4_ through these interactions.

### 3.2. Structural Morphology Analysis of PEI-Fe_3_O_4_

Figure 1c presents the HR-XRD pattern of PEI-Fe_3_O_4_ in a 2θ range of 10° to 80°. The HR-XRD technique is widely used for the structural characterization of crystalline materials. Nine prominent diffraction peaks were observed at 18.36°, 30.19°, 35.52°, 36.50°, 43.17°, 53.66°, 57.18°, 62.83°, and 74.34° corresponding to the Fe_3_O_4_ pattern and showing excellent alignment with the Joint Committee on Powder Diffraction Standards (JCPDS) pattern number 01-088-0315 (Figure 1d). The observed XRD peaks align well with the respective crystallographic planes, such as 18.33° (111), 30.16° (220), 35.52° (311), 37.16° (222), 43.17° (400), 53.56° (422), 57.10° (511), 62.70° (440), and 74.19° (533). The XRD analysis confirms the cubic atomic arrangement and well-ordered crystalline structure of magnetite Fe_3_O_4_ [35,36]. Among all the diffraction peaks, the (311) plane exhibited the highest intensity (3853 counts) and was used for the subsequent crystallite size estimation.

The intensity ratio between the (220) and (311) planes was calculated to be 3.25-fold. The average crystallite size of PEI-Fe_3_O_4_ was estimated using the Scherrer Equation (1).

(1) $$D=\frac{k\lambda }{\beta \times cos\theta }$$

where θ is the Bragg angle, β is the full width at half maximum (FWHM) of the (311) peak (1.94), λ is the X-ray wavelength (0.15406 nm), and k is a constant (~0.9). The average crystallite size of PEI-Fe_3_O_4_ was calculated to be 44.96 nm using the FWHM method [35,36].

**Figure 2:**
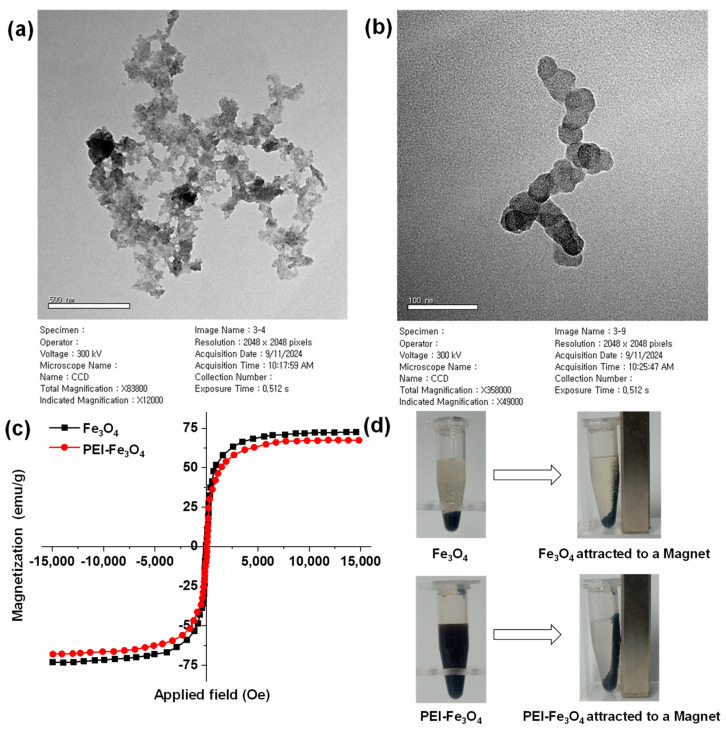
HR-TEM images of PEI-Fe_3_O_4_ taken at (**a**) 500 and (**b**) 100 nm magnifications; (**c**) vibrating sample magnetometry spectra of Fe_3_O_4_ and PEI-Fe_3_O_4_; (**d**) photograph of freshly prepared Fe_3_O_4_ and PEI-Fe_3_O_4_ with external magnet.

Figure 2a and Figure 2b present HR-TEM images of PEI-Fe_3_O_4_ at magnifications of 500 and 100 nm, respectively. Figure S3 a and b show HR-TEM images of PEI-Fe_3_O_4_ taken at 1 µm and 500 nm, respectively. PEI-Fe_3_O_4_ exhibits a spherical morphology with cluster-like assemblies, as observed in the HR-TEM images. In several regions, short chain-like arrangements are also visible, which are likely caused by magnetic dipole–dipole interaction between PEI-Fe_3_O_4_ NPs. The ImageJ software (version 1.54K program, National Institutes of Health, MD, USA) was used to determine the average particle diameter, which was found to be 30.08 nm. The measured particle size was aligned with the crystallite size from the HR-XRD analysis, which was calculated to be approximately 44.96 nm. The findings from HR-XRD and HR-TEM analyses indicate the nanosize, spherical morphology, cubic atomic arrangement, and crystallite nature of the formed PEI-Fe_3_O_4_ [37].

### 3.3. Magnetic Properties of Fe_3_O_4_ and PEI-Fe_3_O_4_

Magnetic properties of the Fe_3_O_4_ and PEI-Fe_3_O_4_ samples were analyzed using the VSM technique at 28 °C. Figure 2c shows the resulting hysteresis (M–H) curves. The values of the remanent magnetization (Mr), coercivity (Hc), and saturation magnetization (Ms) derived from the VSM spectra are listed in Table S2. The experimental magnetic moment in Bohr magnetons (μB) was calculated using Equation (2).

(2) $$\mu B=\frac{Ms\times Mw}{5585}$$

where 5585 is the magnetic factor, M_s_ is the saturation magnetization, and M_w_ is the molecular weight of the sample.

The M_s_ for the Fe_3_O_4_ sample was observed as 72.58 emu/g, relevant to a magnetic moment of 2.28 μB. In comparison, the PEI-Fe_3_O_4_ sample exhibited a slightly reduced M_s_ of 67.39 emu/g, with a magnetic moment of 2.12 μB. Figure 2d displays photographs of freshly prepared Fe_3_O_4_ and PEI-Fe_3_O_4_ samples attracted to an external magnet. Figure S4 shows a photograph of 12-month-aged Fe_3_O_4_ and PEI-Fe_3_O_4_ samples under the same conditions. Even after, 12-month-aged samples retained their original color and strong magnetic properties. However, the Fe_3_O_4_ and PEI-Fe_3_O_4_ samples retained high magnetic moments, which are consistent with those previously reported. These results confirm that the magnetic properties of PEI-Fe_3_O_4_ are minimally affected by the presence of PEI [36,38].

### 3.4. DNA Extraction from E. faecium

**Figure 3:**
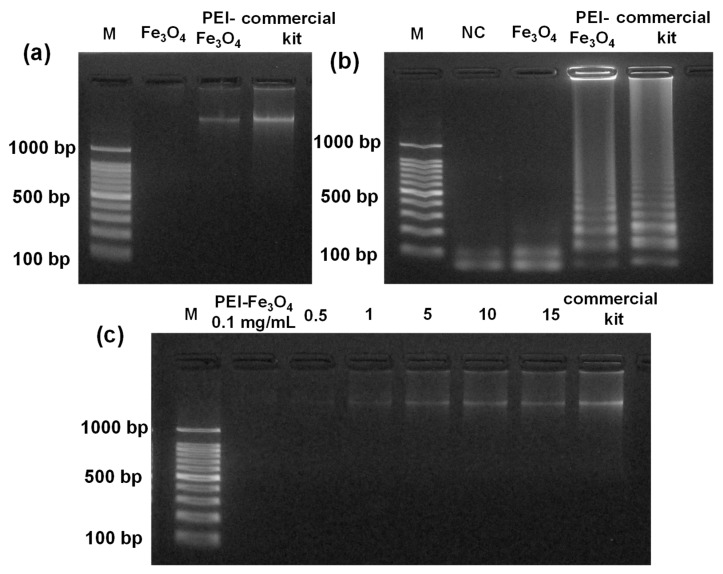
(**a**) Agarose gel electrophoresis of DNA extracted from *E. faecium* using Fe_3_O_4_, PEI-Fe_3_O_4_, and a commercial kit; (**b**) agarose gel electrophoresis of LAMP products obtained using the DNA extracted using 10 mg/mL of Fe_3_O_4_, PEI-Fe_3_O_4_, and a commercial kit; (**c**) gel electrophoresis of DNA extracted from *E. faecium* using PEI-Fe_3_O_4_ at varying concentrations, 0.1, 0.5, 1, 5, 10, and 15 mg/mL, and a commercial kit.

Section 2.5 describes the procedure for isolating DNA from *E. faecium* samples using Fe_3_O_4_, PEI-Fe_3_O_4_, and a commercial kit. Figure 3a displays the gel electrophoresis image of DNA extracted from *E. faecium* using Fe_3_O_4_, PEI-Fe_3_O_4_, and a commercial kit. The gel image showed no visible DNA band for Fe_3_O_4_, whereas strong, high-molecular-weight DNA bands were observed for PEI-Fe_3_O_4_ and the commercial kit, indicating effective extraction by both methods. Figure 3b shows the gel electrophoresis results of LAMP products resulting from DNA extracted using Fe_3_O_4_, PEI-Fe_3_O_4_, and a commercial kit. The gel image showed positive amplification for LAMP-DNA obtained from commercial kit, whereas no amplification observed from LAMP-DNA extracted from Fe_3_O_4_. PEI assists in breaking down bacterial cell walls at 40 °C, whereas the positive charge of PEI-Fe_3_O_4_ attracts negatively charged DNA, thereby facilitating efficient lysis and extraction. Figure 3c shows the agarose gel electrophoresis results of DNA extracted from *E. faecium* using PEI-Fe_3_O_4_ at various concentrations (15, 10, 5, 1, 0.5, and 0.1 mg/mL), along with DNA obtained using a commercial kit. The intensity of the DNA bands increased with high concentration of PEI-Fe_3_O_4_, reaching a maximum at 10 and 15 mg/mL, where the bands showed similar intensity. These results indicate that 10 mg/mL of PEI-Fe_3_O_4_ is sufficient to achieve effective DNA extraction from 250 µL of *E. faecium* culture (6×10^6^ CFU/mL).

Figure S5 shows the absorbance spectra of DNA extracted from *E. faecium* using materials such as Fe_3_O_4_ (10 mg/mL), PEI-Fe_3_O_4_ (0.1, 0.5, 1, 5, 10, and 15 mg/mL), and a commercial kit. The DNA concentration was determined by measuring the absorbance at 260 nm. Figure S5a shows that the DNA extracted with Fe_3_O_4_ exhibited a peak at 235 nm, indicating protein contamination and incomplete cell lysis. Conversely, the absorbance spectra of the PEI-Fe_3_O_4_ samples and the commercial kit did not exhibit the protein peak at 235 nm, thereby confirming the effective cell lysis achieved using PEI-Fe_3_O_4_. The DNA concentrations increased progressively with higher amounts of PEI-Fe_3_O_4_, yielding 4.8, 7.8, 13.2, 15.1, 17.4, and 17.7 ng/µL, respectively. By comparison, the commercial kit yielded higher DNA concentration (29.5 ng/µL) (Figure S5 b–h). The DNA yield remained consistent at 10 and 15 mg/mL of PEI-Fe_3_O_4_, demonstrating that 10 mg/mL is a suitable concentration for efficient DNA extraction. The PEI-Fe_3_O_4_ system achieved an extraction efficiency of 59% relative to the commercial kit. Based on 250 µL of *E. faecium* culture at 6 × 10^6^ CFU/mL (1.5 × 10^6^ cells; ~2 fg DNA per cell), the theoretically calculated DNA is 3.0 ng. A total DNA of 1.77 ng was recovered (17.7 ng/mL in a 100 µL eluate), corresponding to an absolute efficiency of 59%. These results suggest that the PEI-Fe_3_O_4_ system may serve as a cost-effective alternative for DNA extraction.

Figure S6 shows agarose gel electrophoresis of LAMP products obtained using DNA extracted with Fe_3_O_4_ NPs coated with different concentrations of PEI (50, 10, 5, 1, 0.3, and 0.1%). Successful amplifications were observed with 0.1 and 0.3% PEI, as evidenced by the characteristic ladder-like LAMP band pattern, whereas higher PEI concentration (≥1%) inhibited amplifications due to strong PEI-DNA complex formation, which limited template accessibility. Based on these results, 0.3% PEI (PEI-Fe_3_O_4_) is chosen as the optimal concentration, as it enabled cell lysis and DNA extraction without inhibiting the LAMP reaction.

### 3.5. PTA-AuNPs Used as a Colorimetric Probe for LAMP Detection

**Figure 4:**
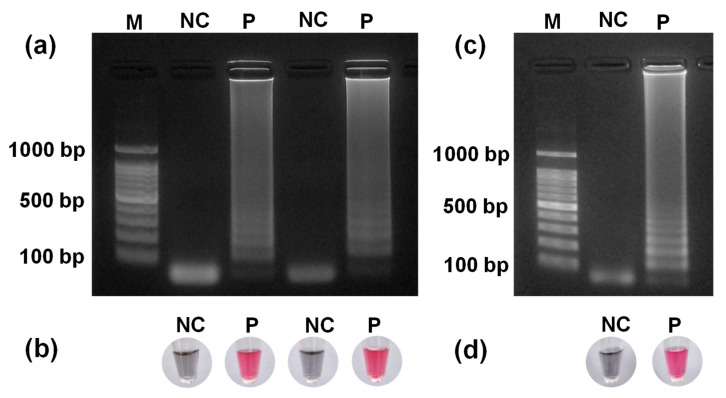
(**a**) Gel electrophoresis of LAMP products obtained using DNA extracted with PEI-Fe_3_O_4_; (**b**) corresponding colorimetric detection of LAMP products using PTA-AuNPs; (**c**) gel electrophoresis of LAMP products obtained using DNA extracted using a commercial kit; (**d**) corresponding colorimetric detection of LAMP products (NC: negative control; P: positive sample).

Figure 4a shows the results of gel electrophoresis for LAMP products from DNA from PEI-Fe_3_O_4_. The gel image shows LAMP-NC and P products performed with DNA at 10 ng/µL. Both lanes corresponding to lane P showed multiple intense DNA bands, confirming successful amplification and demonstrating the reproducibility of the LAMP assay under identical conditions. By contrast, the NC lanes did not show any DNA bands, confirming the absence of amplification. Figure 4b shows the colorimetric visualization of LAMP-amplified DNA using a mixture of HAuCl_4_ and TA at pH 8.5. Figure 4b shows that the P sample appears red, indicating well-dispersed PTA-AuNPs stabilized by the LAMP-amplified DNA. By contrast, the NC appeared gray because of the NPs’ aggregation from insufficient DNA. These results show that the LAMP reaction works consistently at a DNA concentration of 10 ng/µL. PTA-AuNPs enable the clear visual detection of LAMP-amplified DNA by color change, whereas gel electrophoresis and color detection together provide a reliable method to detect *E. faecium* DNA.

Figure 4c shows the gel electrophoresis results of LAMP products obtained using DNA extracted with the commercial kit, whereas Figure 4d presents the colorimetric detection of these LAMP products using a mixture of HAuCl_4_ and TA at pH 8.5. The gel image shows NC and LAMP reactions containing 10 ng/µL of DNA. In lane P, multiple intense bands confirmed successful DNA amplification. The PTA-AuNPs provided clear visual cues, with red indicating the P sample and gray indicating the NC sample. The gel images and corresponding color changes with DNA extracted using the commercial kit and PEI-Fe_3_O_4_ showed consistent results across both methods.

### 3.6. Results of the Sensitivity and Selectivity Assay

PTA-AuNPs were used to detect *E. faecium* plasmid DNA based on color changes produced by LAMP-amplified DNA. Serial dilutions of *E. faecium* DNA were prepared, ranging from 10 ng/μL down to 0.1 fg/μL. Figure S7 shows the gel electrophoresis results for *E. faecium* DNA across the aforementioned concentration range. Gel electrophoresis revealed a strong DNA band at the highest concentration tested (10 ng/μL). At lower concentrations (0.1 fg/μL), no bands were visible, indicating that conventional gel electrophoresis has limited sensitivity for detecting lower amounts of DNA. LAMP combined with colorimetric assays using PTA-AuNPs improved the detection of low DNA concentrations.

**Figure 5:**
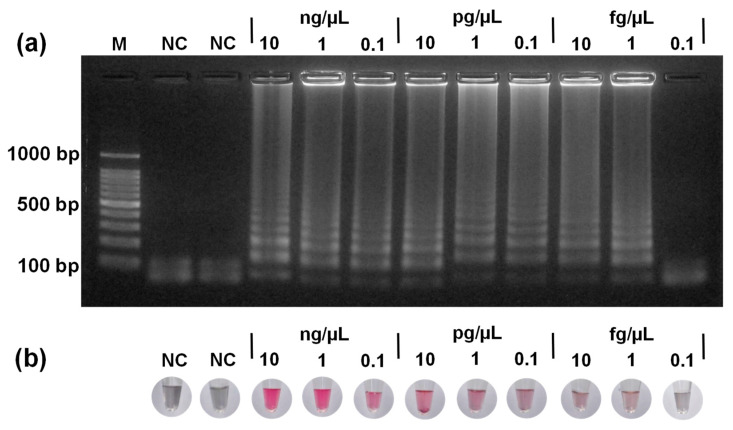
(**a**) Gel electrophoresis of LAMP products for DNA concentrations ranging from 10 ng/μL down to 0.1 fg/μL, along with the NC; (**b**) corresponding colorimetric detection of LAMP products using PTA-AuNPs.

Figure 5a shows the gel electrophoresis of LAMP-amplified DNA for the sensitivity study, displaying a similar ladder-like pattern of bands from 10 ng/µL to 1 fg/µL, indicating that amplification was successful across the respective concentration range. Figure 5b shows the corresponding colorimetric detection of LAMP-amplified DNA using PTA-AuNPs. Red color was observed in samples with DNA concentrations between 10 ng/µL and 1 fg/µL, confirming successful LAMP. The intensity of the red color gradually decreased as the DNA concentration decreased, reflecting lower levels of amplification. By contrast, the samples with 0.1 fg/μL and the NC appeared gray, indicating ineffective LAMP. These colorimetric results were consistent with the gel electrophoresis results. The colorimetric results indicated that the PTA-AuNPs system could detect LAMP-amplified DNA concentrations as low as 1 fg/µL (LOQ). Overall, this method offers a rapid and sensitive alternative to conventional gel electrophoresis for detecting LAMP-amplified DNA.

Figure S8 shows gel electrophoresis results demonstrating the selectivity of the LAMP assay. The samples included NC, *E. faecium* (Efm), *S. pneumonia* (Spn), and their mixture (*E. faecium* + *S. pneumonia*). The gel images confirmed successful amplification of LAMP products from 10 ng/µL of *E. faecium* DNA alone, as well as from a mixture containing 10 ng/µL of *E. faecium* DNA and 10 ng/µL of *S. pneumonia* DNA. In contrast, no amplification was observed for 10 ng/µL of *S. pneumonia* DNA alone, indicating that the designed LAMP primers selectively targeted *E. faecium* without cross-interferences.

### 3.7. Smartphone-Based Detection of LAMP-Amplified DNA

**Figure 6:**
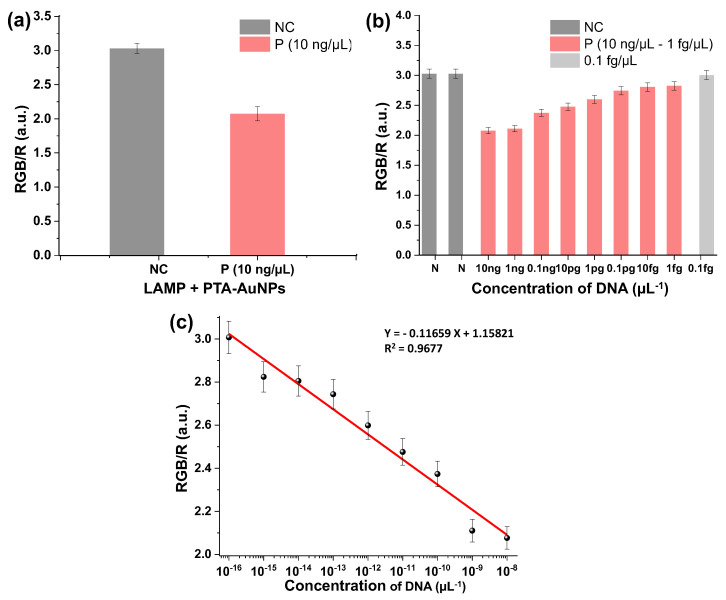
(**a**) RGB/R ratio of colorimetric LAMP results for NC and P samples; (**b**) RGB/R ratios for different LAMP-amplified DNA concentrations (0.1 fg/µL to 10 ng/µL); (**c**) linear plot showing the relationship between DNA concentration and RGB/R ratio.

Section 2.11 outlines the procedure for detecting the LAMP-amplified DNA using smartphone-based RGB colorimetric analysis. Colorimetric images of the LAMP assay with PTA-AuNPs were captured using a 200-megapixel smartphone camera (Figure 5b). Colorimetric images were analyzed for RGB values using the Color Picker application. Figure S9 shows screenshots from the Color Picker application utilized for RGB-based quantitative analysis. Figure S9a presents the interface of an NC sample characterized by gray coloration, whereas Figure S9b depicts a P sample exhibiting a distinct red shift, which is indicative of successful LAMP. Figure 6a presents a bar graph of the RGB/R ratios obtained from the PTA-AuNPs-based colorimetric LAMP assay, highlighting the distinct colorimetric differences between the NC and P samples, as indicated by variations in their respective RGB values. Figure 6b displays a bar graph of RGB/R ratios for NC and P samples across DNA concentrations ranging from 0.1 fg/µL to 10 ng/µL. The NC sample exhibited a high RGB/R ratio, whereas the values for P samples progressively decreased with increasing DNA concentration (1 fg/µL to 10 ng/µL), demonstrating a clear concentration-dependent colorimetric response. Figure 6c shows a linear correlation between DNA concentration and the RGB/R ratio, exhibiting strong linearity (R^2^ = 0.9677) over the range of 0.1 fg/µL to 10 ng/µL. Overall, the results confirm that smartphone-based RGB/R analysis enables the accurate quantification of LAMP-amplified DNA. Compared with commercial kits (3 h, $5/test), the PEI–Fe_3_O_4_/PTA–AuNPs system completes extraction and detection in 70 min at $1.5/test, demonstrating rapid and cost-effective performance. Specifically, the workflow consists of 10 min for DNA extraction, 45 min for LAMP, and 15 min for smartphone and colorimetric detection. Furthermore, commercial kits rely on multiple centrifugation steps and harsh chaotropic salts, whereas the PEI–Fe_3_O_4_/PTA–AuNPs system eliminates these requirements by integrating bacterial lysis, magnetic DNA capture, and colorimetric detection into an efficient workflow.

### 3.8. Practical Application on Real Samples

**Figure 7:**
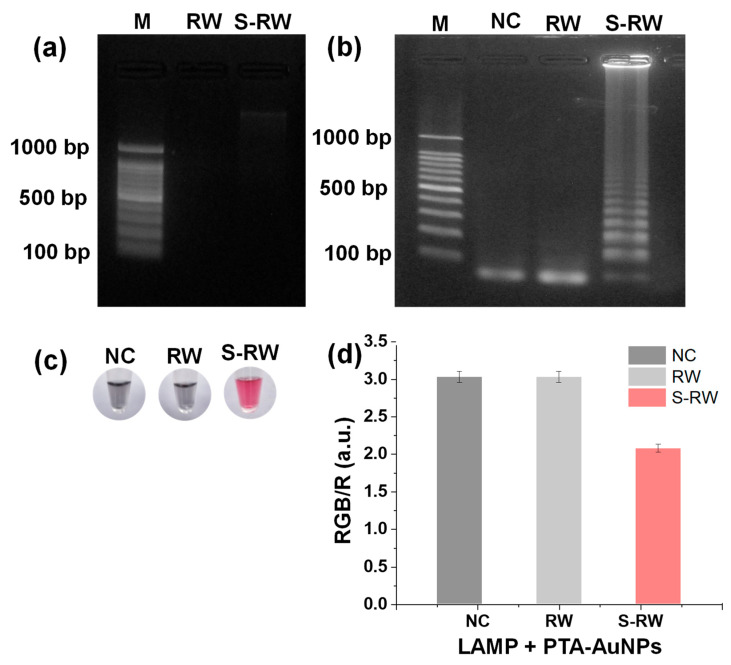
(**a**) Gel electrophoresis of DNA extracted from river water (RW) and *E. faecium*-spiked RW (S-RW) using PEI-Fe_3_O_4_; (**b**) gel electrophoresis of LAMP-amplified DNA products from RW and S-RW samples; (**c**) corresponding colorimetric detection of LAMP mixtures for NC, RW, and S-RW samples using PTA-AuNPs; (**d**) bar graph of RGB/R ratios obtained from PTA-AuNPs-based analysis of LAMP mixtures for NC, RW, and S-RW samples.

NAT, including DNA extraction, LAMP, and colorimetric detection, was carried out on real environmental samples to demonstrate the practical utility of the PEI-Fe_3_O_4_/PTA-AuNPs hybrid system. The river water (RW) sample underwent an initial pretreatment by centrifugation to remove large solid impurities, followed by filtration using Whatman filter paper to eliminate fine particulate matter. The resulting clarified RW sample was then analyzed for DNA extraction using PEI-Fe_3_O_4_, facilitating efficient DNA isolation. Figure 7a displays gel electrophoresis results of DNA extracted from RW and 6 × 10^6^ CFU/mL of *E. faecium*-spiked RW (S-RW) using PEI-Fe_3_O_4_, demonstrating the effectiveness of the magnetic extraction method. Figure S10 shows the absorbance spectra of DNA extracted from the RW and S-RW samples. No detectable DNA concentration was observed in the unspiked RW, whereas the S-RW sample showed a measurable DNA concentration of 10 ng/µL.

The DNA extracted from RW and S-RW samples was used for LAMP. As shown in Figure 7b, gel electrophoresis of the LAMP products confirmed the successful amplification of the S-RW sample, whereas no amplification was observed in the RW sample. Figure 7c presents the corresponding colorimetric detection using PTA-AuNPs. A distinct red color was observed in the LAMP products of the S-RW sample, whereas the RW and NC samples exhibited a gray color, indicating the absence of DNA amplification. Figure 7d shows a bar graph of the RGB/R ratios obtained from PTA-AuNPs mixed with the LAMP products of the NC, RW, and S-RW samples. The significant difference in the RGB/R values between the RW and S-RW samples confirmed the successful colorimetric detection of the amplified DNA. Overall, the magnetic-nanogold hybrid system integrated into the NAT workflow provides a rapid, cost-effective, and portable approach for the extraction and detection of *E. faecium* DNA from RW samples. However, the magnetic-nanogold hybrid system has some limitations, including lower DNA yield (59%) than commercial kits, use of an external magnet, and involvement of multiple steps. Future improvements will aim to enhance yield, reduce workflow complexity, and advance toward a true POCT diagnostic platform.

## 4. Conclusions

In this study, a novel PEI-Fe_3_O_4_/PTA-AuNPs hybrid system was developed to enable NAT, including cell lysis with DNA extraction, isothermal amplification, and naked-eye detection, thereby providing a rapid, cost-effective, and portable approach for bacterial analysis. Fe_3_O_4_ and PEI-Fe_3_O_4_ were synthesized via an ultrasonication-assisted co-precipitation method. The optical, structural, and magnetic properties of Fe_3_O_4_ and PEI-Fe_3_O_4_ were characterized using UV–Vis spectroscopy, FT-IR spectroscopy, HR-XRD, HR-TEM, and VSM. The magnetic PEI-Fe_3_O_4_ was employed to efficiently extract DNA from *E. faecium* within 10 min at 40 °C, yielding a DNA concentration of 17.4 ng/µL. This extraction protocol achieved 59% efficiency compared with that of a commercial kit (29.5 ng/µL), highlighting its effectiveness and cost-saving potential. The extracted DNA was used for LAMP at 65 °C for 45 min. The PTA-AuNPs were used as colorimetric probes for the visual detection of LAMP-amplified DNA. A distinct red-to-gray color change was observed between P and NC samples, with a LOQ as low as 1 fg/µL. Finally, smartphone-based RGB analysis was used to quantify the colorimetric signal, enabling a simple, equipment-free, on-site readout. Our PEI–Fe_3_O_4_PTA–AuNPs system achieves DNA extraction and detection within 70 min at a cost of only $1.5 per test. Overall, the magnetic-nanogold hybrid system facilitated DNA extraction and colorimetric LAMP detection, making it well-suited for microbial monitoring.

## Data Availability

Data are contained within the article.

## Supplementary Materials

The following supporting information can be downloaded at https://www.mdpi.com/article/10.3390/bios15090601/s1, Figure S1: schematic of the synthesis of iron oxide (Fe_3_O_4_) and polyethyleneimine-functionalized Fe_3_O_4_ (PEI-Fe_3_O_4_); Figure S2: schematic representation of the integrated nucleic acid testing protocol using PEI-Fe_3_O_4_ and poly(tannic acid)-stabilized gold nanoparticles (PTA-AuNPs); Table S1: target genes and corresponding primer sequences used in the loop-mediated isothermal amplification (LAMP) assay; Figure S3: HR-TEM images of PEI-Fe_3_O_4_ at (a) 1 µm and (b) 500 nm magnifications; Table S2: vibrating sample magnetometry-derived magnetic properties of Fe_3_O_4_ and PEI-Fe_3_O_4_; Figure S4: photograph of 12-month-aged Fe_3_O_4_ and PEI-Fe_3_O_4_ with external magnet; Figure S5: nanodrop absorbance spectra of DNA extracted from *E. faecium* using methods such as (a) Fe_3_O_4_ (10 mg/mL); (b) to (g) PEI-Fe_3_O_4_ at 0.1, 0.5, 1, 5, 10, and 15 mg/mL; and (h) a commercial kit; Figure S6: agarose gel electrophoresis of LAMP products obtained using *E. faecium* DNA extracted with Fe_3_O_4_ nanoparticles coated with different concentrations of PEI (50, 10, 5, 1, 0.3, and 0.1%); Figure S7: agarose gel electrophoresis of serially diluted *E. faecium* DNA samples ranging from 10 ng/µL to 0.1 fg/µL; Figure S8: agarose gel electrophoresis showing the selectivity of the LAMP products for the negative control (NC), *E. faecium* (Efm), *Streptococcus pneumoniae* (Spn), and their mixture (Efm + Spn); Figure S9: screenshot of the Color Picker application used for LAMP detection with PTA-AuNPs (a) NC and (b) positive sample; Figure S10: nanodrop absorbance spectra of DNA extracted using PEI-Fe_3_O_4_ from (a) river water (RW) and (b) *E. faecium*-spiked RW.
