# Supplementary material for: PEI-Fe3O4/PTA-AuNPs Hybrid System for Rapid DNA Extraction and Colorimetric LAMP Detection of E. faecium
Source: Biosensors (Basel). 2025 Sep 12;15(9):601. doi: 10.3390/bios15090601 (PMC12467045; doi:10.3390/bios15090601)
Supplement: Supplementary file 1 [file biosensors-15-00601-s001.zip › biosensors-3802696-supplementary.pdf]

Supporting Information for

PEI-Fe_3O_4/PTA-AuNPs Hybrid System for Rapid DNA Extraction and Colorimetric LAMP Detection of *E. faecium*

Muniyandi Maruthupandi [1], Haang Seok Choi [2], and Nae Yoon Lee [2,*]

[1] Department of BioNano Convergence, Gachon University, 1342 Seongnam-daero, Sujeong-gu, Seongnam-si 13120, Gyeonggi-do, Republic of Korea; maruthu1328@gachon.ac.kr (M.P.)
[2] Department of BioNano Technology, Gachon University, 1342 Seongnam-daero, Sujeong-gu, Seongnam-si 13120, Gyeonggi-do, Republic of Korea; choihas@gachon.ac.kr (H.S.C.)
* Correspondence: nylee@gachon.ac.kr

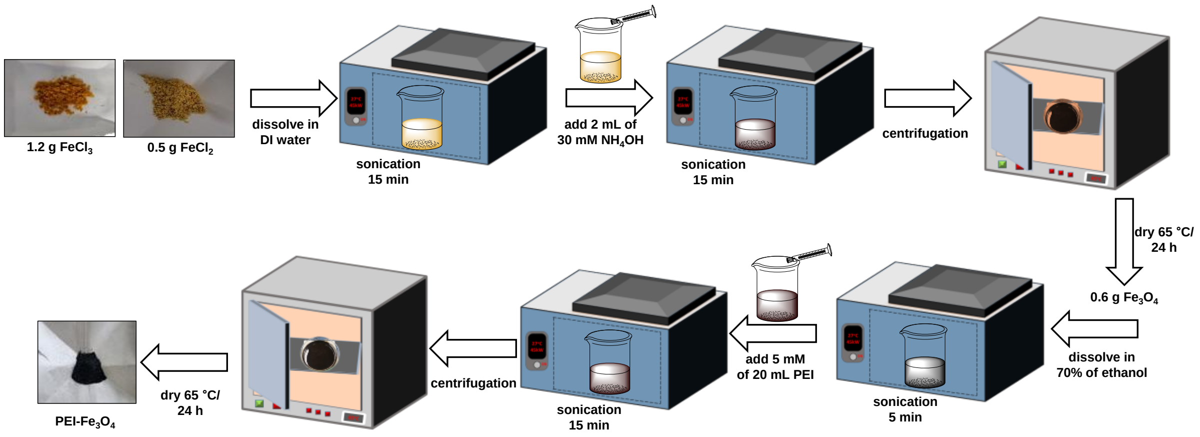

Figure S1: Schematic of the synthesis of iron oxide (Fe_3O_4) and polyethyleneimine functionalized Fe_3O_4 (PEI-Fe_3O_4).

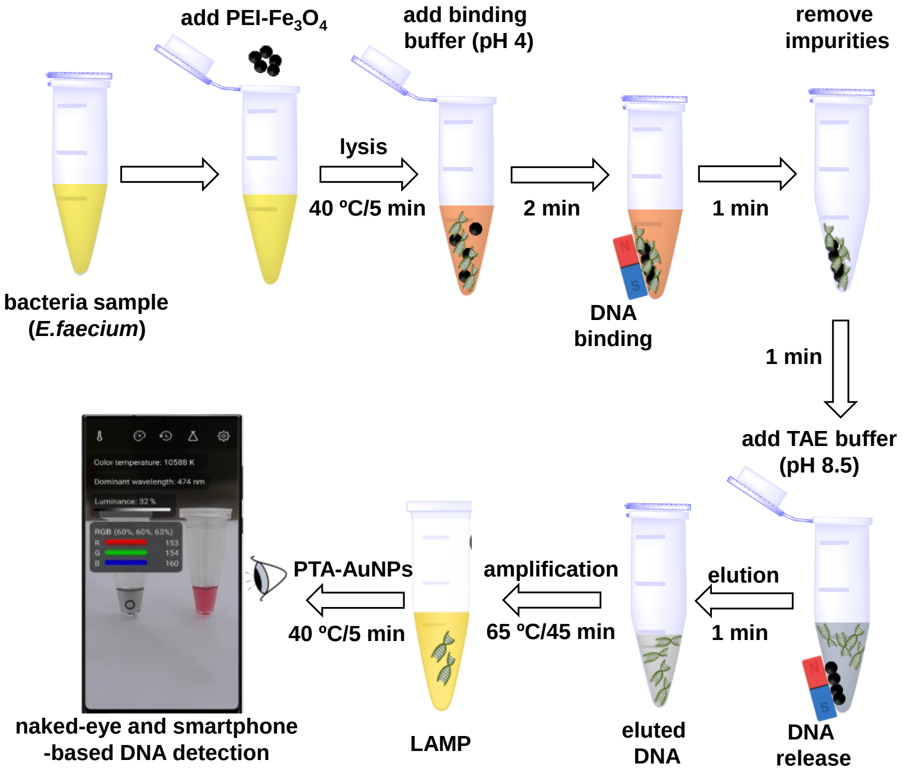

Figure S2: Schematic representation of the integrated nucleic acid testing protocol using PEI-Fe_3O_4 and poly(tannic acid)-stabilized gold nanoparticles (PTA-AuNPs).

Table S1: Target genes and corresponding primer sequences used in the loop-mediated isothermal amplification (LAMP) assay.

Target Gene	Sl. No.	Primer	Primer Sequences (5′′-3′)′
esp gene (*E. faecium*)	1	LB	TGA TGT TGA CAC AAC AGT TAA GGG
	2	F3	CCA GAA CAC TTA TGG AAC AG
	3	B3	GTT GGG CTT TGT GAC CTG
	4	FIP	CGT GTC TCC GCT CTC TTC TTT TTA TTT GCA AGA TAT TGA TGG TG
	5	BIP	ATC GGG AAA CCT GAA TTA GAA GAA GAA CTC GTG GAT GAA TAC TTT C

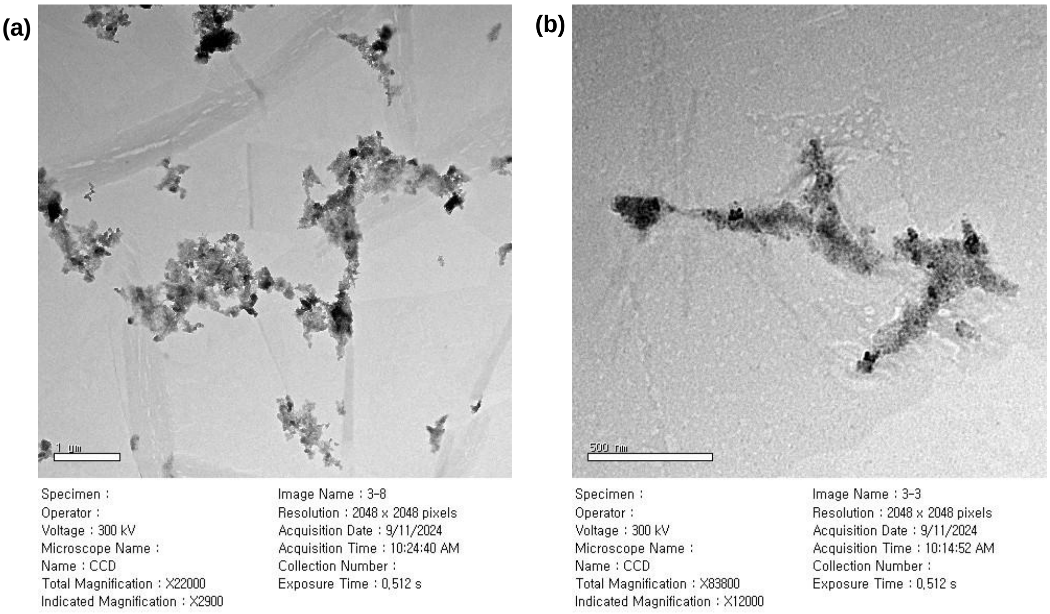

Figure S3: HR-TEM images of PEI-Fe_3O_4 at **(a)** 1 μm and **(b)** 500 nm magnifications.

Table S2: Vibrating sample magnetometry-derived magnetic properties of Fe_3O_4 and PEI-Fe_3O_4.

Sl.No.	Sample	Hc (Oe)	Ms (emu/g)	Mr (emu/g)	Bohr magneton (μB)
1	Fe_3O_4	51.77	72.58	8.03	2.28
2	PEI-Fe_3O_4	129	67.39	9.75	2.12

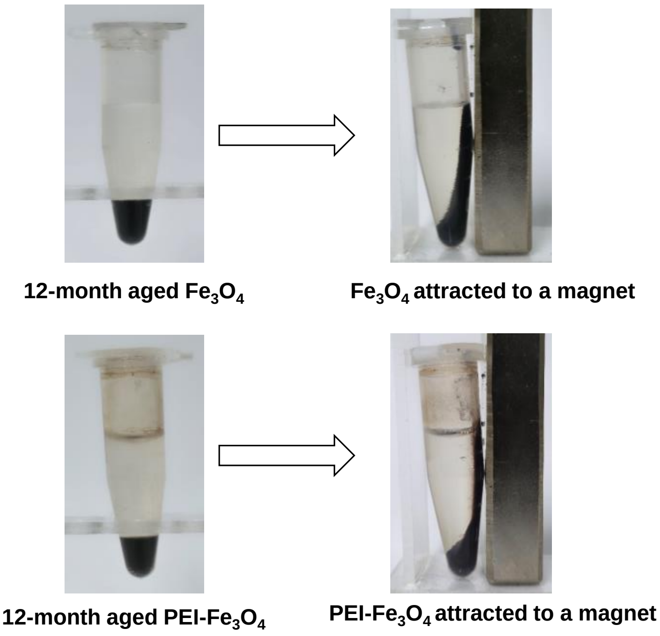

Figure S4: Photograph of 12-month aged Fe_3O_4 and PEI-Fe_3O_4 with external magnet.

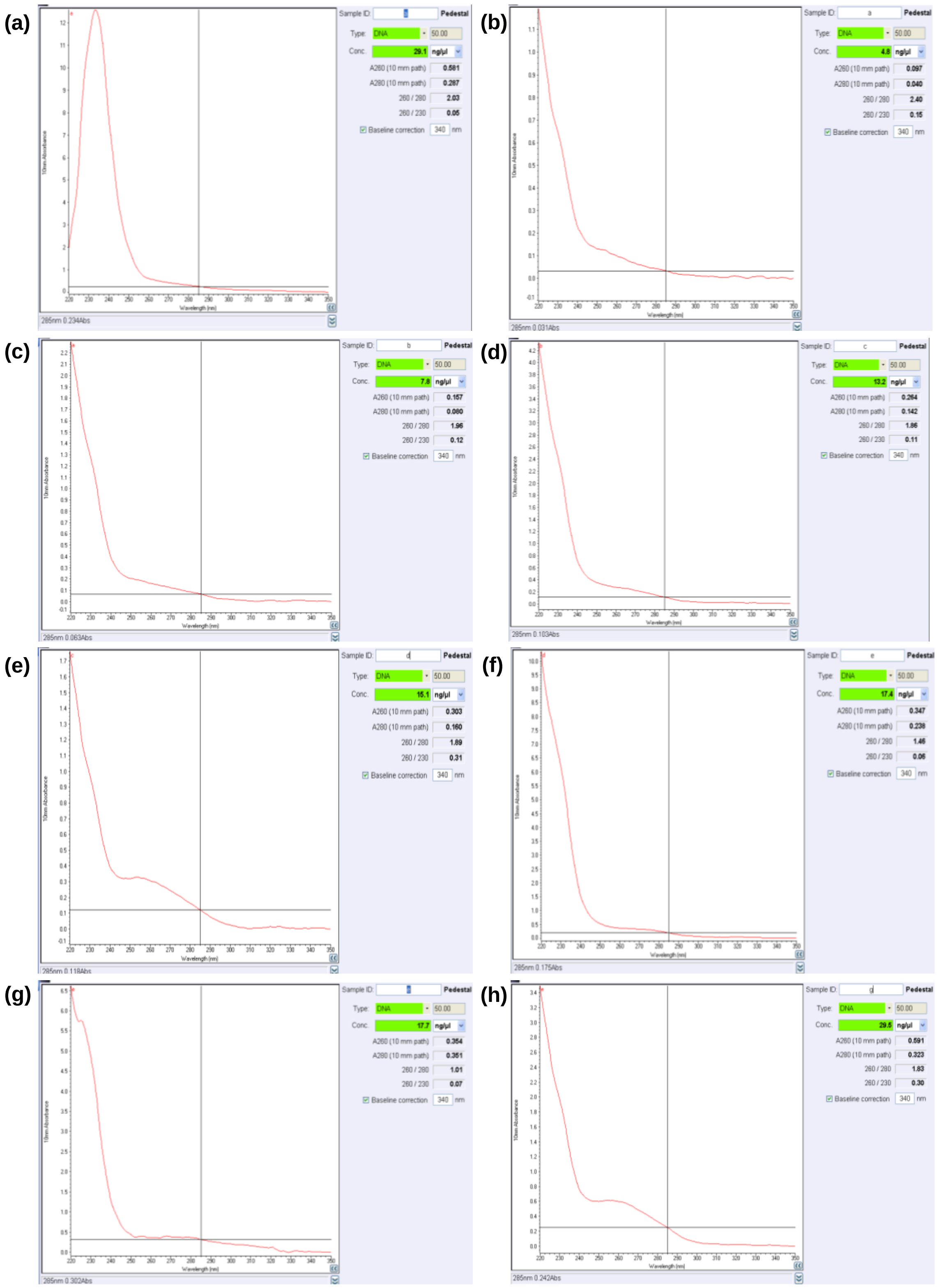

Figure S5: Nanodrop absorbance spectra of DNA extracted from *E. faecium* using methods such as **(a)** Fe_3O_4 (10 mg/mL); **(b)** to **(g)** PEI-Fe_3O_4 at 0.1, 0.5, 1, 5, 10, and 15 mg/mL; and **(h)** a commercial kit.

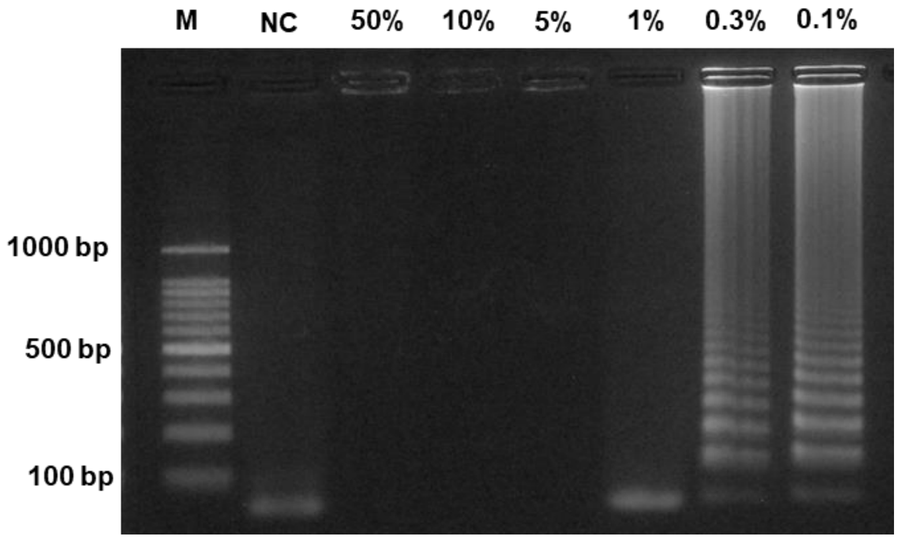

Figure S6: Agarose gel electrophoresis of LAMP products obtained using *E. faecium* DNA extracted with Fe_3O_4 nanoparticles coated with different concentrations of PEI (50, 10, 5, 1, 0.3, and 0.1%).

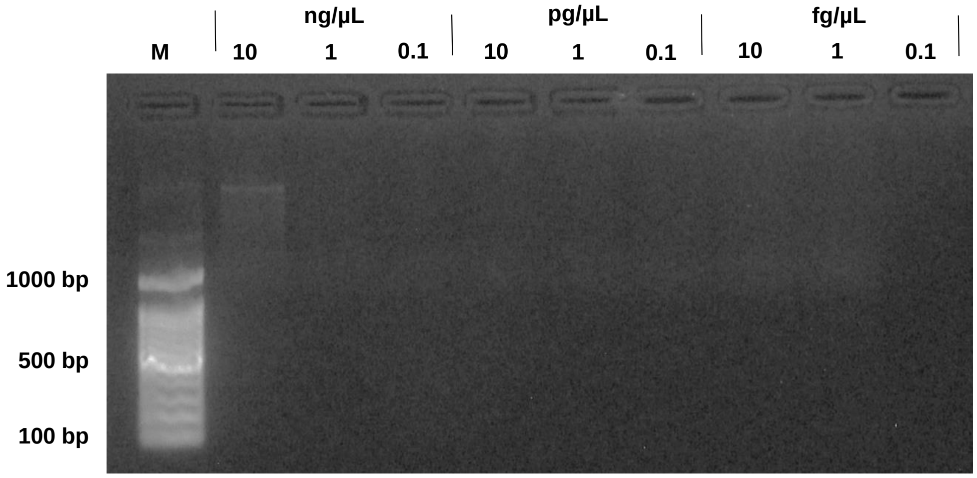

Figure S7: Agarose gel electrophoresis of serially diluted *E. faecium* DNA samples ranging from 10 ng/µL to 0.1 fg/µL.

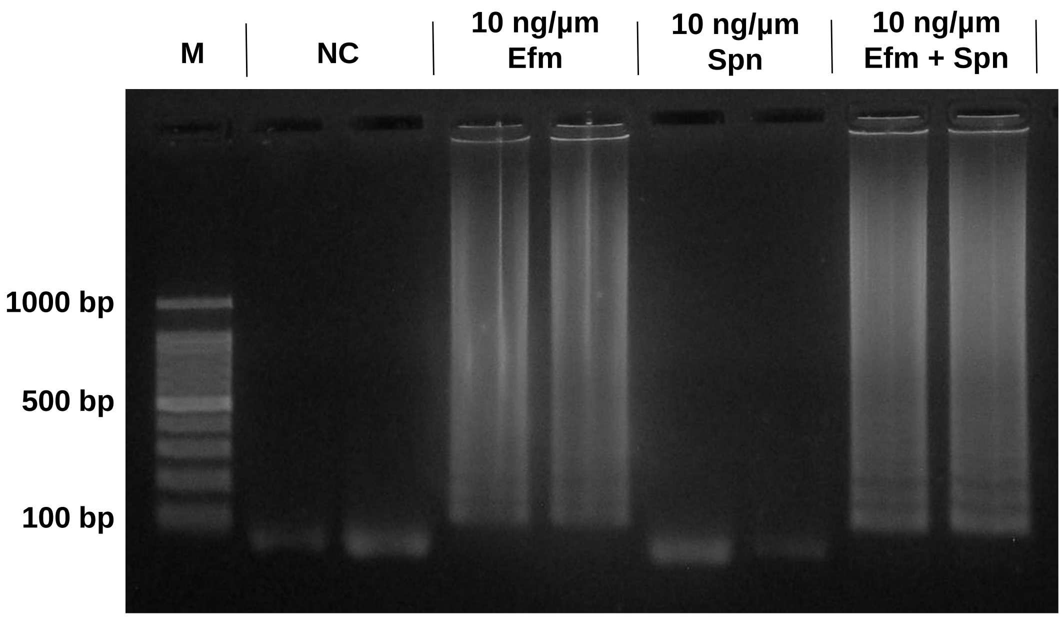

Figure S8: Agarose gel electrophoresis showing the selectivity of the LAMP products for the negative control (NC), *E. faecium* (Efm), *Streptococcus pneumoniae* (Spn), and their mixture (Efm + Spn).

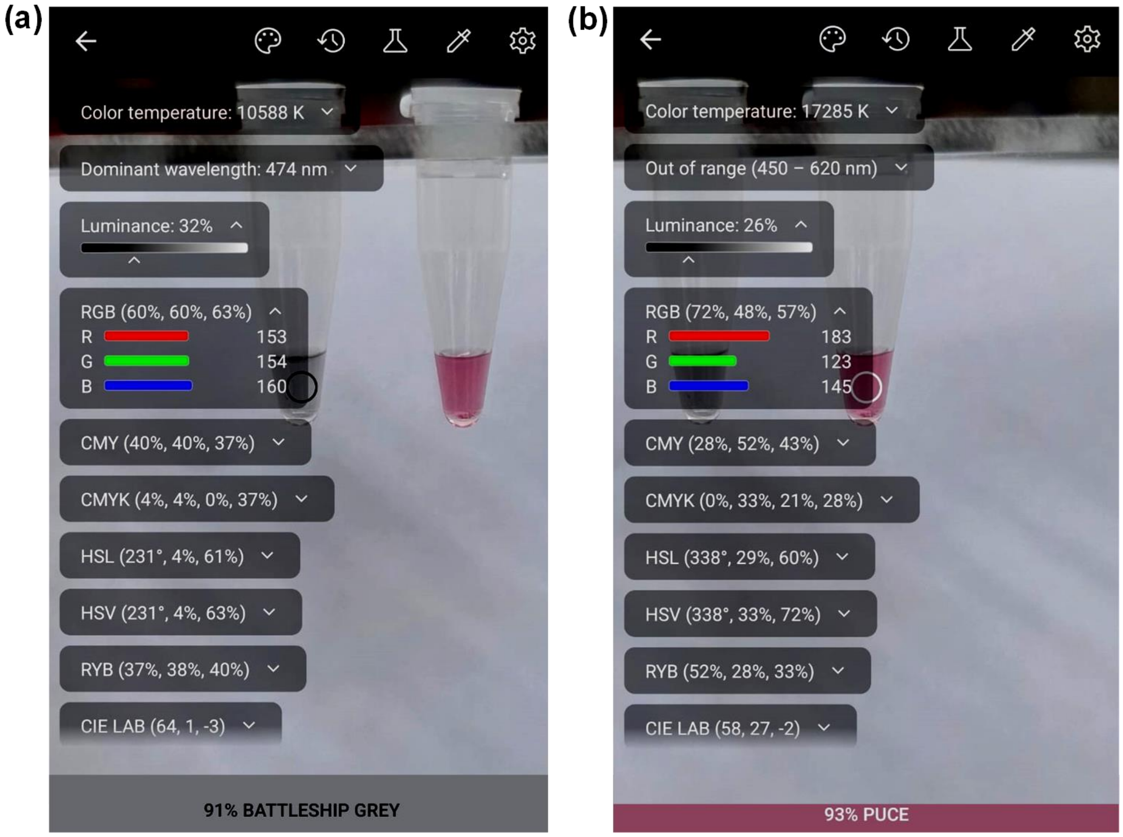

Figure S9: Screenshot of the Color Picker application used for LAMP detection with PTA-AuNPs **(a)** NC and **(b)** positive sample.

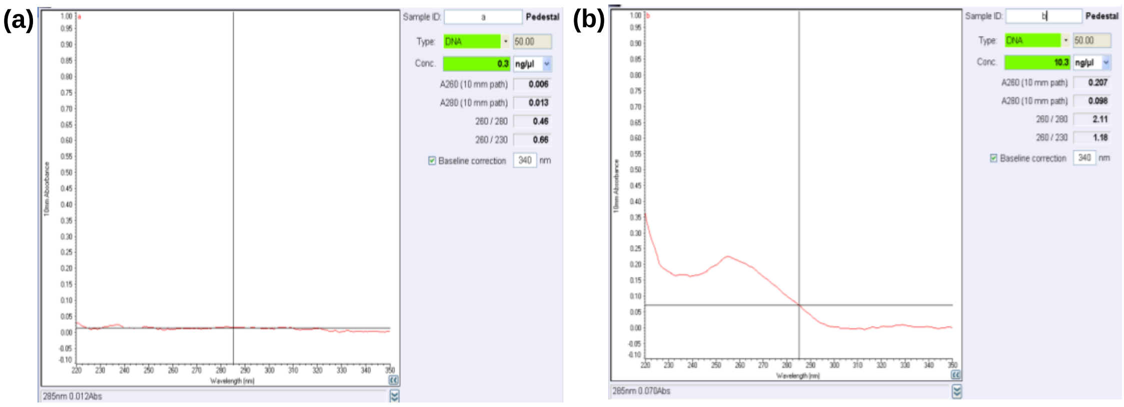

Figure S10: Nanodrop absorbance spectra of DNA extracted using PEI-Fe_3O_4 from **(a)** river water (RW) and **(b)** *E. faecium*-spiked RW.
